# Inhibition of nitrogen fixation in symbiotic *Medicago truncatula* upon Cd exposure is a local process involving leghaemoglobin

**DOI:** 10.1093/jxb/ert334

**Published:** 2013-10-22

**Authors:** Daniel Marino, Isabelle Damiani, Sébastien Gucciardo, Iker Mijangos, Nicolas Pauly, Alain Puppo

**Affiliations:** ^1^Interactions Biotiques et Santé Végétale UMR INRA 1301 – CNRS 6243 – Université de Nice – Sophia Antipolis, 400 Route des Chappes, BP 167, F-06903 Sophia Antipolis Cedex, France; ^2^Department of Plant Biology and Ecology, University of the Basque Country (UPV/EHU), Apdo. 644, E-48080, Bilbao, Spain; ^3^Ikerbasque, Basque Foundation for Science, E-48011, Bilbao, Spain; ^4^NEIKER-Tecnalia, Basque Institute of Agricultural Research and Development, E-48160 Derio, Spain

**Keywords:** Antioxidant, cadmium, *Medicago truncatula–Sinorhizobium meliloti* symbiosis, nitrogen fixation, reactive oxygen species, split-root system.

## Abstract

Leguminous biological nitrogen fixation (BNF) is very sensitive to environmental fluctuations. It is still contentious how BNF is regulated under stress conditions. The local or systemic control of BNF and the role played by reactive oxygen species (ROS) in such regulation have still not been elucidated completely. Cadmium, which belongs to the so-called heavy metals, is one of the most toxic substances released into the environment. The mechanisms involved in Cd toxicity are still not completely understood but the overproduction of ROS is one of its characteristic symptoms. In this work, we used a split-root system approach to study nodule BNF and the antioxidant machinery’s response to the application of a mild Cd treatment on one side of a nodulated *Medicago truncatula* root system. Cd induced the majority of nodule antioxidants without generating any oxidative damage. Cd treatment also provoked BNF inhibition exclusively in nodules directly exposed to Cd, without provoking any effect on plant shoot biomass or chlorophyll content. The overall data suggest that the decline in BNF was not due to a generalized breakdown of the plant but to control exerted through leghaemoglobin/oxygen availability, affecting nitrogenase function.

## Introduction

Legumes have the capacity to use atmospheric nitrogen (N_2_) as a nitrogen source so they can fulfil their nitrogen demand. This ability comes from the symbiotic relationship between legumes and soil bacteria that leads to the formation of root nodules, where biological nitrogen fixation (BNF) takes place (Oldroyd *et al.*, 2011). However, the yield of legume crops is often limited by the extreme sensitivity of BNF to environmental fluctuations (Zahran, 1999). Several factors, including oxygen availability and plant carbon and nitrogenous status, are linked to BNF inhibition under abiotic stresses, but the mechanism responsible for the inhibition remains largely unknown. Carbon status might be regulated by either the shoot through photosynthesis inhibition or a direct carbon shortage in the nodules due to inhibition of sucrose synthase (González *et al.*, 1995). Oxygen availability can be regulated by different factors including the O_2_-binding protein leghaemoglobin (Lb) or the variable oxygen diffusion barrier (ODB) in the nodule endodermis, which maintains the microaerobic environment within the infected zone of the nodules (Minchin, 1997; Minchin *et al.*, 2008). One open question is whether the origin of BNF inhibition comes from direct inhibition within the nodules or as systemic feedback signalling from the shoot (Serraj *et al.*, 2001; Marino *et al.*, 2007; Gil-Quintana *et al*., 2013*a*, 2013*b*).

Cadmium, which belongs to the so-called heavy metals, is one of the most toxic substances released into the environment. It mainly enters the environment as a consequence of human activity from industrial processes and by the use of fertilizers and pesticides (Verbruggen *et al*,. 2009; Clemens *et al.*, 2013). Exposure to Cd, even at low concentrations, can lead to irreversible cell damage in virtually every living organism (Verbruggen *et al*,. 2009; Clemens *et al.*, 2013). Some effects of Cd accumulation in plants include leaf chlorosis, root necrosis, and a decrease in plant growth (Gallego *et al.*, 2012). The mechanisms involved in Cd toxicity are still not completely understood but the overproduction of reactive oxygen species (ROS) is one of its associated characteristic symptoms (Cuypers *et al.*, 2010).

ROS, such as hydrogen peroxide, singlet oxygen, and superoxide anion, are constantly produced in association with aerobic life (Apel and Hirt, 2004). In nodules, high respiration rates—together with high concentrations of Lb and the abundance of catalytic Fe—enhance, among other things, nodule capacity to generate ROS (Marino *et al.*, 2009). Under stress conditions ROS play a dual role: they may act as both signal transduction molecules and toxic agents oxidizing cellular macromolecules (Apel and Hirt, 2004; Suzuki *et al.*, 2012). Thus, maintaining a redox balance must be strictly controlled to avoid ROS deleterious effects. To do so, plants possess a complex array of detoxification mechanisms including enzymes such as catalases or superoxide dismutases (SODs) and metabolites like glutathione and ascorbate (Apel and Hirt, 2004).

Legume nodules are an excellent means to illustrate the dual signalling and toxicity roles of ROS. For instance, disruption of nodule functionality due to natural ageing or to exposure to severe changes in the environment, like drought, salinity, or darkness, has been suggested to be provoked by the oxidative damage associated with ROS overproduction during these processes (Becana *et al.*, 2010). However, in nodules, ROS also have an important role in signalling processes. For example, the application of a low concentration of paraquat, a ROS generator, provoked BNF inhibition associated with ROS as signalling molecules rather than as deleterious molecules (Marino *et al.*, 2006). Moreover, in *Medicago truncatula* an RNA interference approach directed against *MtRbohA*, a nodule-enhanced NADPH oxidase gene, provoked a decrease in nodule nitrogen-fixation activity and the modulation of genes encoding the microsymbiont nitrogenase (Nase), suggesting a positive role of ROS in proper nodule functioning (Marino *et al.*, 2011). Similarly, an NADPH oxidase of common bean has been shown to be crucial for successful rhizobial colonization (Montiel *et al.*, 2012).

The mechanism underlying ROS generation upon Cd exposure remains to be elucidated. Cd itself is not redox active since it is not able to trigger the Fenton reaction (Salin, 1988), but it has been suggested that Cd induction of ROS production can be related to suppression of antioxidant defences (Sandalio *et al.*, 2001), to disruption of the electron transport chain, and somehow to the activation of plasma membrane NADPH oxidases (Romero-Puertas *et al.*, 2004; Cuypers *et al.*, 2010). It has been widely reported that Cd impairs nodule functioning associated with photosynthesis and chlorophyll depletion and to nodule oxidative damage in soybean (Balestrasse *et al.*, 2006), alfalfa (Shvaleva *et al.*, 2010), white lupin (Carpena *et al.*, 2003), and mung bean (Muneer *et al.*, 2012), among other legumes. Moreover, it has been suggested that resistance or minor sensibility to Cd depends on the plant’s ability to increase its antioxidant defences (Noctor and Foyer, 1998; Paradiso *et al.*, 2008).

However, little is known about the cause of the impairment of nodule function under mild stress and the potential relationship of the induction of ROS production with this BNF inhibition. Therefore, the aim of this work was to better understand nitrogen-fixation regulation under abiotic stress. We used the model legume *M. truncatula* grown in a split-root-system, in which only one side of a nodulated root was exposed to Cd. This system allowed us to uncouple the direct effect of Cd on nodules from the consequences derived from a systemic effect of Cd on the aerial part of the plant. Moreover, this approach was useful to investigate how nodule metabolism and antioxidant machinery are modulated under these conditions and their relationship with BNF inhibition. This work represents, to our knowledge, the first time that the effects of Cd on *M. truncatula* nodule physiology have been studied.

## Materials and methods

### Plant growth and Cd treatment

**Fig. 1.**
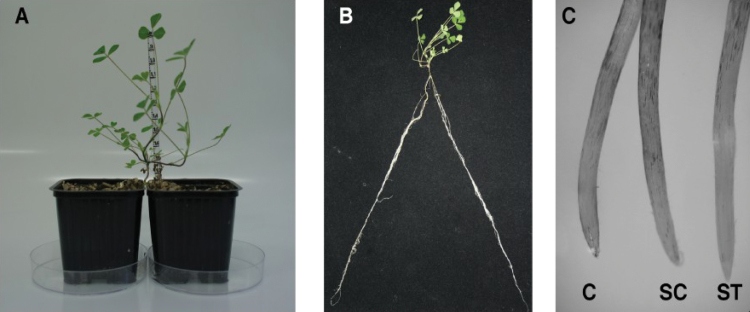
*M. truncatula* plants grown in split-root-system (A) leading to a root system divided into two equal parts (B). Evans blue staining of *M. truncatula* secondary root apex to detect cell death after Cd treatment (C). This figure is available in colour at JXB online.

*M. truncatula* cv Jemalong J6 was used throughout the experiments. Surface-sterilized seeds were placed on 0.4% agar plates in the dark for 24h at 4 °C and then for 3 days at 14 °C. The root tip of germinated seeds was removed and plants were transferred into modified Farhaeus agar plates to induce the production of new roots. Ten days later plants were transferred into a double pot of 2×250ml with a 1:2 sand/vermiculite mixture in a controlled environment chamber (25/22 °C day/night; 75% hygrometry; 200 µE·m^−2^·s^−1^ light intensity and 16/8h light/dark photoperiod), and watered with a nitrogen-free nutrient solution (Rigaud and Puppo, 1975). Each side of the root system was inoculated with a *Sinorhizobium meliloti* 2011 strain. Five weeks after the transfer, plants were divided randomly in two groups: one control (C) and one treatment (T). For T plants, one pot was daily irrigated at field capacity with nutrient solution supplemented with 1mM CdCl_2_ while in the other pot the nutrient solution was supplemented with 1mM CaCl_2_ (Fig. 1A, B). For C plants both pots were daily irrigated at field capacity with nutrient solution supplemented with 1mM CaCl_2._ Seventy-two hours later plant material was harvested, immediately frozen in liquid nitrogen, and stored at −80 °C.

### Detection of cell death

To determine cell viability under Cd treatment, roots were infiltrated with a 0.25% (w/v) aqueous solution of Evans blue (Rodriguez-Serrano *et al.*, 2006)

### Cd content determination

To analyse Cd content, shoots and nodulated roots were harvested. Roots were thoroughly rinsed with deionized water to eliminate the Cd that could be superficially adsorbed. Plant material was oven-dried at 70 °C for 48h, and the dry weight was recorded. Subsamples (0.4g) of dried plant tissue were digested with a mixture of HNO_3_/HClO_4_ (Zhao *et al.*, 1994). Cd content in the digest was determined using flame atomic absorption spectrometry (Varian, Palo Alto, CA, USA).

### Chlorophyll determination

The content of chlorophyll *a* and *b* was determined spectrophotometrically as described by Arnon (1949) in 80% aqueous acetone extracts by measuring absorbance at 645 and 663nm.

### Nitrogen fixation

Nitrogen fixation was determined through acetylene-reduction assay. Freshly harvested nodulated roots from detopped plants were placed in rubber-capped 30ml glass bottles filled with an acetylene/air mixture (C_2_H_2_/air, 1:10 v/v). After 1h of incubation at 25 °C the amount of ethylene in the gas phase was determined by gas chromatography using a 6890N Network GC system (Agilent Technologies, La Jolla, CA, USA).

### Lipid peroxidation

Lipid peroxidation in nodules was assayed by measuring the concentration of malondialdehyde (MDA), the major 2-thiobarbituric acid-reacting substance (TBARS). Modifications as proposed by Hodges *et al.* (1999) were carried out to correct interference generated by non-specific turbidity, thiobarbituric acid–sugar complexes, and other non-TBARS compounds absorbing at 532nm.

### Total RNA isolation, reverse transcription, and gene-expression analysis

Nodules were homogenized in liquid nitrogen with a mortar and pestle and total RNA extraction and reverse transcription were assayed as described in Andrio *et al.* (2013). Quantitative real-time PCR was carried out using the qPCR Mastermix Plus for SYBR Green I reagent (Eurogentec, Angers, France). Reactions were run on the Chromo4 Real-Time PCR Detection System (Bio-Rad, Hercules, CA, USA) and quantification was performed with Opticon Monitor analysis software version 3.1 (Bio-Rad). Every reaction was set up in three technical replicates. The PCR program used was as follows: polymerase activation (95 °C for 5min), amplification and quantification cycles repeated 40 times (94 °C for 15 s, 60 °C for 1min), and melting curve (40–95 °C with one fluorescence read every 0.5 °C). The mRNA levels were normalized against two endogenous controls: *40S Ribosomal Protein S8* (TC137982) and *Mtc27* (TC132510) (Van de Velde *et al.*, 2006) were used for *M. truncatula* and *Smc00324* and *ribosomal rna16S* housekeeping genes were used to normalize the bacterial mRNA levels (Becker *et al.*, 2004). The following formula was used to calculate the relative expression ratio: 2^−Δ*CT*^, with Δ*CT* = *CT*_gene of interest_−*CT*_houskeeping gene_. For each experiment, stability of the reference genes across samples was tested using geNorm software (Vandesompele *et al.*, 2002). The absence of contamination with genomic DNA was tested by PCR in all RNA samples, prior to reverse transcription, using the primers of the housekeeping genes. The gene-specific primers used are listed in Table S1.

### Protein extraction and enzyme activities

Nodules were homogenized with a mortar and pestle in 50mM Tris/HCl, 0.1mM EDTA, 0.2% Triton X-100, 1mM PMSF, 5 µM E64 (3 µl/ml), and 2mM dithiothreitol (pH 7.8) at 0–2 °C (5ml g^−1^ fresh weight). The homogenate was centrifugated for 30min at 20 000g at 4 °C. An aliquot of the supernatant was retained for plant-fraction protein determination (Bradford, 1976) and the rest was used to measure the following enzyme activities: NADP^+^-dependent isocitrate dehydrogenase (ICDH; EC 1.1.1.42), ascorbate peroxidase (APX; EC 1.11.1.11), catalase (CAT; EC 1.11.1.6), glutathione reductase (GR; EC 1.6.4.2), and unspecific peroxidases (guaiacol peroxidases, GPX; EC 1.11.1.7). ICDH was determined as described by Marino *et al.* (2006), and APX, CAT, GR, and GPX were assayed as described by Zabalza *et al.* (2007).

### Western blot

Sodium dodecyl sulphate/polyacrylamide gel electrophoresis (SDS/PAGE) was performed according to Laemmli (1970) with a 1 mm-thick 12% acrylamide (w/v) resolving gel and a 4.6% (w/v) stacking gel in a vertical electrophoresis cell (Mini- Protean III; Bio-Rad) at 150V for 60min. Gels were electroblotted onto polyvinylidene fluoride membrane for 75min at 100V in a Mini Trans-Blot Electrophoretic Transfer Cell (Bio-Rad). Blots were blocked in 5% (w/v) skim milk in 20mM Tris-buffered saline at 4 °C overnight. We used α-sucrose synthase (SS) (1:5000), α-Lb (1.2000), and α-nitrogenase (1:2000) as primary antibodies. The secondary antibody was goat anti-rabbit horseradish peroxidase conjugate (1:50 000, Sigma-Aldrich). Immunoreactive bands were visualized with a highly sensitive chemiluminescent substrate for peroxidase detection (GE Healthcare Europe GmbH, Freiburg, Germany).

### Statistical analyses

All the data presented are given as means with standard errors. The significance of the results was assessed using multiple-factor analysis of variance (*P* value <0.05).

## Results

### Cd exposure provokes local nitrogen-fixation inhibition without affecting aerial parts of the plant

**Fig. 2.**
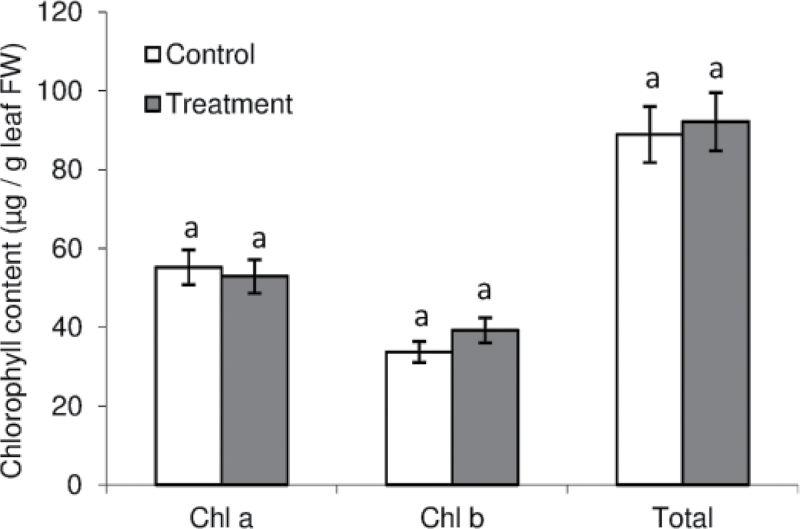
Chlorophyll content in control and Cd-treated *M. truncatula* plants grown in a SRS. Each bar shows mean±SE for *n*=6. FW, fresh weight.

Split-root system (SRS) approaches are very useful to study local or systemic effects of a stress applied on the root system, in this case Cd. When using a SRS approach to study root responses to an environmental change it is suitable that the applied stress provokes a minimum effect on the aerial part for better discrimination between local or systemic effects. To use the lowest Cd dose that in our conditions could lead to nitrogen-fixation impairment, we first screened the effect of different Cd treatments on the Nase activity of *M. truncatula* nodules. We applied six different concentrations of CdCl_2_ (0, 0.1, 0.2, 0.5, 1, and 2mM) for 3 days, after which we selected 1mM, the lowest concentration that in our conditions provoked nitrogen-fixation inhibition (data not shown). Plants in the SRS (Fig. 1A, B) were divided into two groups: one control (C) with both sides of the root system irrigated with nutrient solution and one Cd-treated (T) with one half of the root system treated with Cd (split-treatment, ST) and the other half irrigated with nutrient solution (split-control, SC). Leaf chlorosis, root necrosis, and a decrease in plant growth are some of the most characteristic symptoms of Cd toxicity (Gallego *et al.*, 2012). The aim of our work was to apply a slight Cd stress to avoid these symptoms. Thus, we used these three parameters as indicators of the severity of our treatment. Cd application did not affect shoot biomass (Table 1), nor chlorophyll *a* and *b* content (Fig. 2), indicating that 3 days of Cd exposure did not provoke damage to the aerial part. Root and nodule biomass were similar in ST, SC, and C roots (Table 1) and no root necrosis could be observed as determined by Evans blue staining (Fig. 1C).

**Table 1.** Biomass (fresh weight) of control and Cd-treated M. truncatula nodules grown in SRS. Root and nodule fresh weight values of control plants correspond to the mean of half roots

	Shoot (g)	Root (g)	Nodules (mg)
C	0.74±0.05	0.56±0.04	25.67±3.02
SC		0.66±0.05	25.94±4.11
ST		0.58±0.03	24.41±2.57
T	0.75±0.04		

Each value represents mean±SE for *n*=8.

**Fig. 3.**
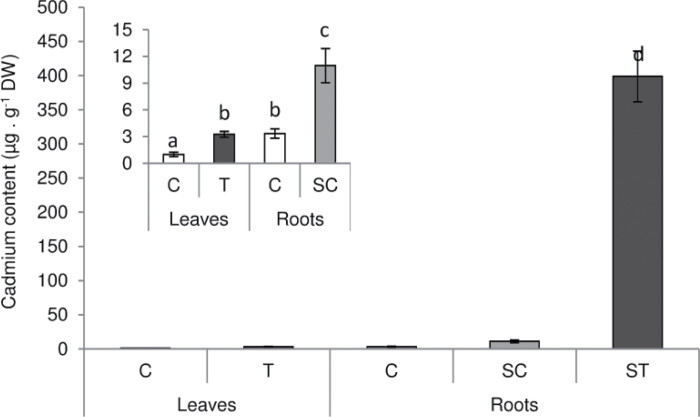
Cd content in leaves and roots of control and Cd-treated *M. truncatula* plants grown in a SRS. Each bar represents mean±SE for *n*=8. Different letters represent significant differences (*P*≤0.05). The inset shows the detail for C, T, and SC plants. DW, dry weight.

Cd content in ST roots was close to 400 µg g^−1^ dry weight, about 100-fold higher than in C roots (Fig. 3). Cd can enter the roots through symplastic and apoplastic pathways and is translocated via the vascular system to the rest of the plant (Mendoza-Cózatl *et al.*, 2011). In this work, at the time of harvest, only approximately 1% of the Cd taken up by ST roots was translocated to shoots or SC roots (Fig. 3).

**Fig. 4.**
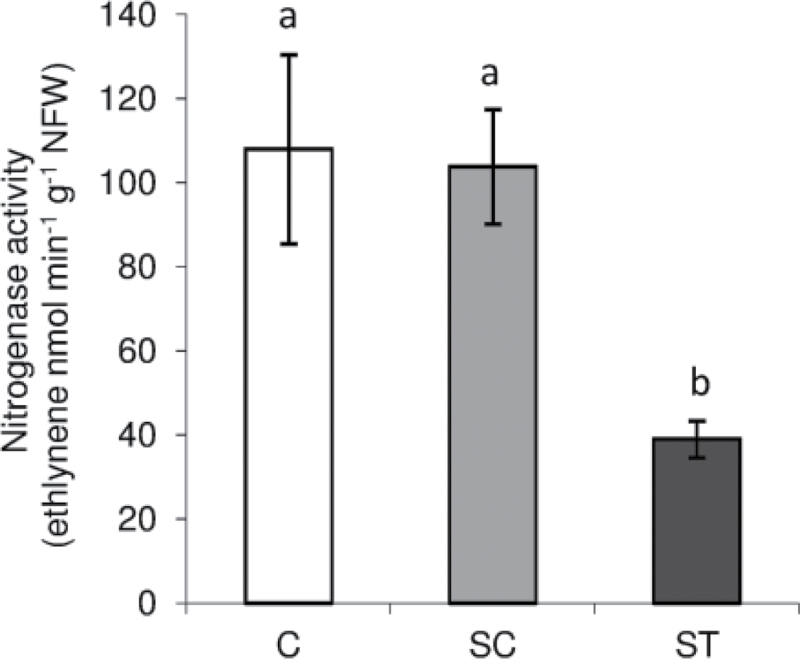
Nase activity of control and Cd-treated *M. truncatula* nodules grown in a SRS. Each bar represents mean±SE for *n*=8. Different letters represent significant differences (*P*≤0.05). NFW, nodule fresh weight.

Nase activity in ST nodules was dramatically reduced while the activity in SC nodules was similar to control plants (Fig. 4). This result shows that nitrogen fixation activity under Cd stress is locally controlled. It has been previously shown that acetylene may inhibit Nase activity and that it can affect the functioning of the ODB (Minchin *et al.*, 1983, 1986). In this work BNF was determined by the traditional acetylene-reduction assay. The limitations associated with this technique must be taken into account to interpret the approximately 60% BNF decline in ST nodules compared to SC nodules not as a quantitative measurement but as a rather qualitative one, showing that BNF was inhibited by Cd treatment only in ST nodules.

### Cd up-regulates nodule antioxidant machinery

**Fig. 5.**
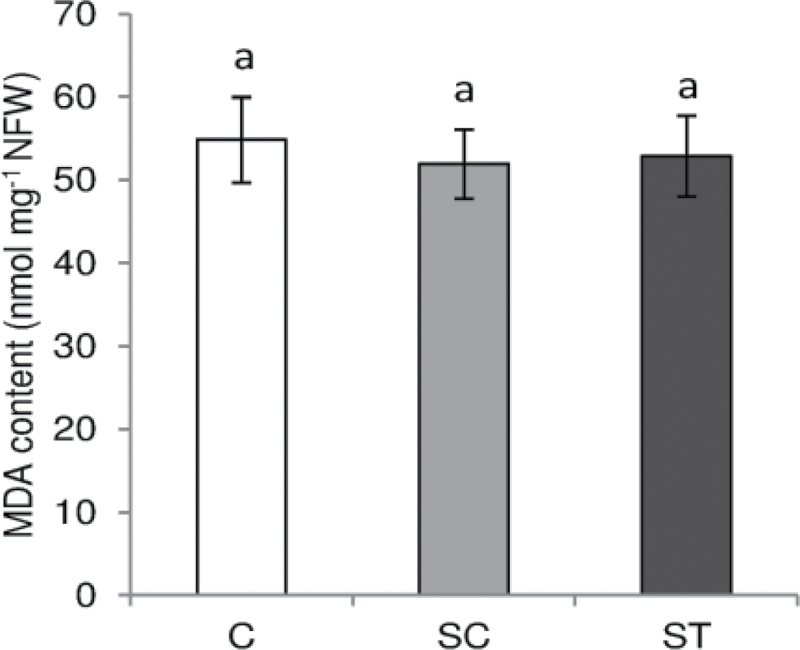
MDA content of control and Cd-treated *M. truncatula* nodules grown in a SRS. Each bar represents mean±SE for *n*=6. Different letters represent significant differences (*P*≤0.05). NFW, nodule fresh weight.

Cd is widely known to generate lipid oxidation (Cuypers *et al.*, 2010). Thus, we measured lipid oxidation through the determination of MDA content as a marker of oxidative damage in nodules. No significant modification in MDA content was observed in ST nodules compared to SC and C nodules (Fig. 5). According to this result, Cd did not provoke a generalized oxidative damage in nodules at the time of harvest.

**Fig. 6.**
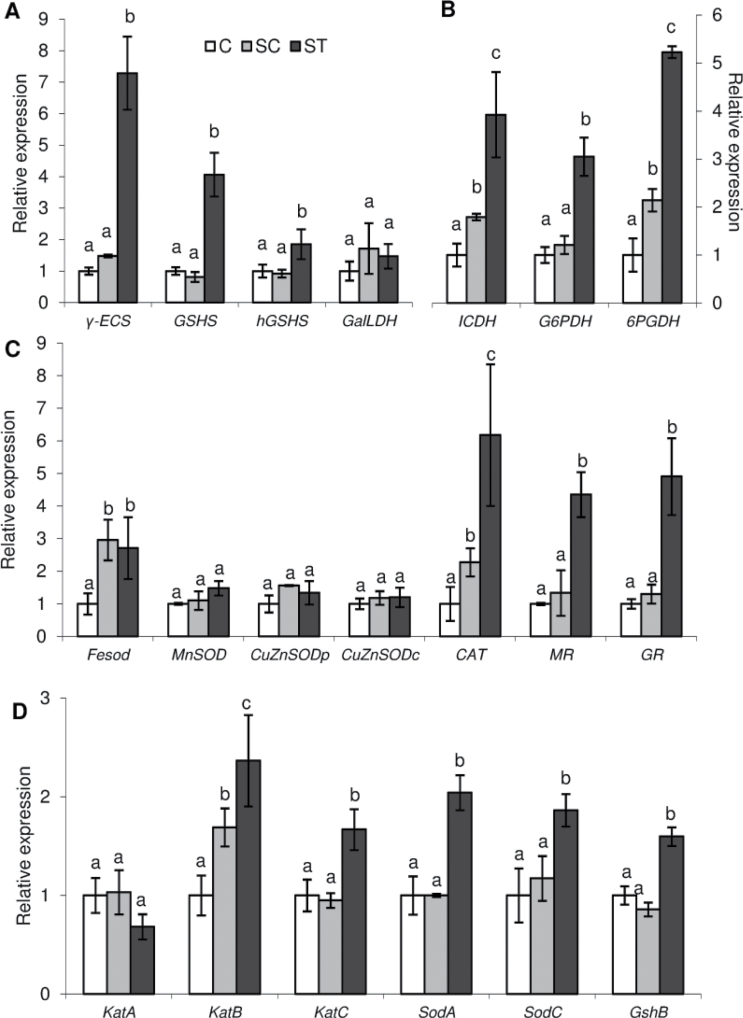
Expression quantification by quantitative PCR of genes encoding (A) plant components of non-enzymatic antioxidant machinery, (B) plant NADPH-generating enzymes, (C) plant antioxidant enzymes, and (D) bacterium antioxidant components. Each bar represents mean±SE for *n*=6. Different letters represent significant differences (*P*≤0.05). GSHS, glutathione synthetase; hGSHS, homoglutathione synthetase; GalLDH, galactono-1,4-lactone dehydrogenase; G6PDH, glucose-6-phosphate dehydrogenase; 6PGDH, 6-phosphogluconate dehydrogenase.

To investigate how nodular antioxidant defence responded to Cd and whether its control was local or systemic we determined by quantitative PCR the expression of genes encoding some of the nodule main antioxidant machinery components, from both the plant and the bacterial partners (Fig. 6). For the plant side, these include SODs, a family of metalloenzymes harbouring Fe, Mn, or CuZn in the active site that detoxify O_2_^−^ to H_2_O_2_, and CAT, an enzyme that directly detoxifies H_2_O_2._ We also investigated components of non-enzymatic antioxidant biosynthesis such as γ-glutamyl-cysteine synthetase (γ-ECS), glutathione synthetase and homoglutathione synthetase for thiol synthesis, and galactono-1,4-lactone dehydrogenase, the enzyme catalysing the last step in the main pathway of ascorbate biosynthesis. We also analysed the expression of genes encoding monodehydroascorbate reductase (MR) and GR, enzymes of the ascorbate-glutathione cycle. Moreover, NADPH recycling has been described as a factor limiting plant antioxidant capacity (Foyer and Noctor, 2009). Thus, we determined the expression of genes encoding the main NADPH-generating enzymes which include ICDH and the pentose-phosphate pathway NADP^+^-generating glucose-6-phosphate dehydrogenase and 6-phosphogluconate dehydrogenase. From the microsymbiont we analysed the gene expression of bacterial antioxidants including SODs (*SodA*, *SodC*), catalases (*KatA*, *KatB*, *KatC*), and glutathione synthetase (*Gshb*).

On the plant side, the three genes of (homo)glutathione synthesis were locally up-regulated by Cd (Fig. 6A). Among these three genes, γ-ECS presented the highest induction, about seven times in ST nodules compared to C and SC nodules (Fig. 6A). Similarly, the three genes encoding NADPH-generating enzymes were induced in ST nodules (Fig. 6B). Interestingly, ICDH and 6-phosphogluconate dehydrogenase were slightly induced in SC compared to C nodules (Fig. 6B). Among the genes encoding antioxidant enzymes, *FeSOD*, *CAT*, *MR*, and *GR* were induced in ST nodules compared to C nodules (Fig. 6C). Among them, *MR* and *GR* were exclusively induced in ST nodules whereas *CAT* was partially induced in SC nodules compared to C nodules (Fig. 6C). *FeSOD* was the unique gene that experienced an induction in SC nodules similar to that of ST nodules upon Cd exposure (Fig. 6C). At the bacterial level *KatC*, *SodA*, *SodC*, and *Gshb* were locally induced and *KatB* was induced in both SC and ST nodules (Fig. 6D).

**Fig. 7.**
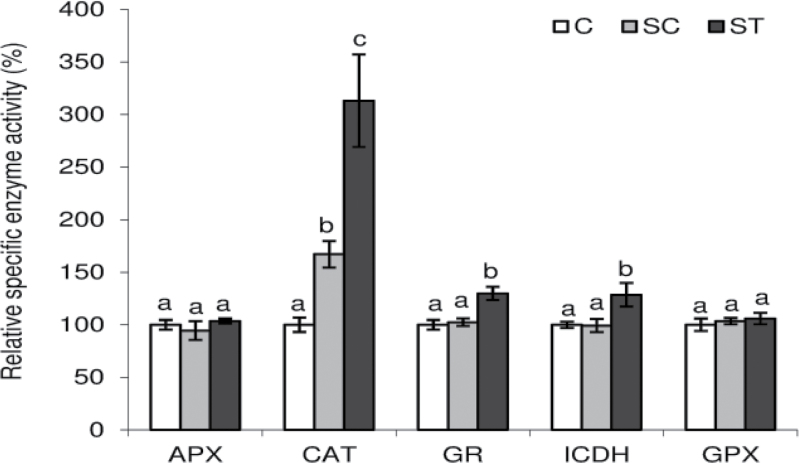
Enzyme activity of *M. truncatula* nodule APX, CAT, GR, ICDH, and GPX. Each bar represents mean±SE for *n*=6. Different letters represent significant differences (*P*≤0.5).

We also determined the nodule plant fraction enzyme activities of APX, CAT, GR, ICDH, and unspecific peroxidases (GPX). GR and ICDH activities were locally induced (Fig. 7). In agreement with its gene expression, CAT activity was enhanced both in SC and ST nodules but the induction was 2-fold higher in the nodules directly exposed to Cd (ST). GPX and APX activities did not respond to Cd (Fig. 7).

### Cd effect on nitrogen fixation is related to Lb and Nase down-regulation

**Fig. 8.**
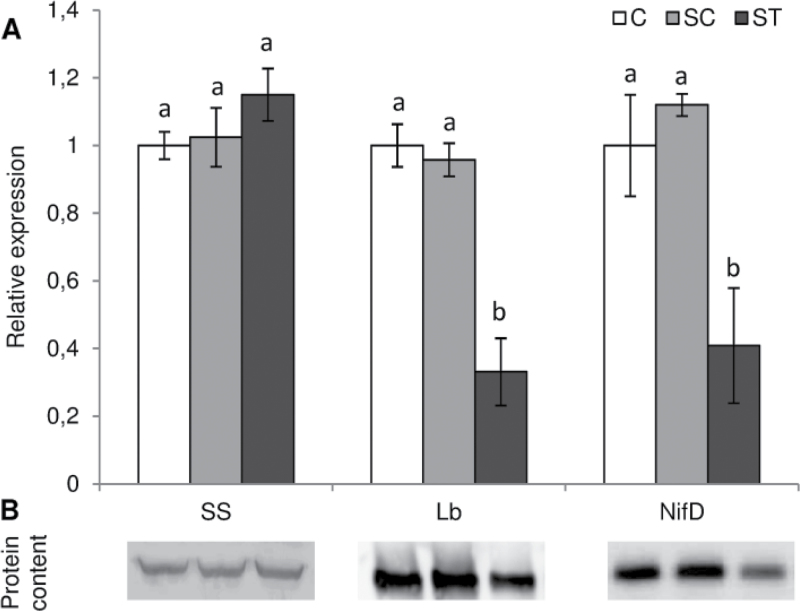
Gene expression (A) and protein content (B) of SS, Lb, and a Nase component (NifD) of control and Cd-treated *M. truncatula* nodules grown in a SRS. Each bar represents mean±SE for *n*=6. Different letters represent significant differences (*P*≤0.05).

To gain further knowledge on the potential responsible agents of nitrogen fixation activity decline we determined by quantitative PCR the expression of genes encoding some of the main actors of nodule response to abiotic stresses: SS, Lb, and the Nase component NifD. The *SS* expression level was similar in every treatment. However both *Lb* and *NifD* expression declined locally in ST nodules (Fig. 8A). We also determined by Western blot the protein amounts of SS, Lb, and NifD with specific antibodies (Fig. 8B). In accordance with gene-expression levels, SS protein content did not vary while Lb and NifD declined locally in the nodules directly exposed to Cd (Fig. 8B).

## Discussion

The decline of nitrogen fixation under abiotic stress has been related to a shortage of carbon due to a reduction of plant photosynthetic capacity or to a direct shortage in the nodule, related to down-regulation of SS, the enzyme that catalyses the first step of sucrose hydrolysis before entering the glycolytic pathway (González *et al.*, 1995). There is also evidence, mainly obtained in legumes of tropical origin, that BNF inhibition is due to feedback control related to plant ‘nitrogenous status’ (King and Purcell, 2005). Finally, it has been shown that oxygen is a key factor in BNF regulation, related both to the need for oxygen in obtaining energy and to ROS damage of nodule components such as Lb or Nase (Becana *et al.*, 2010). Moreover, Lb oxidation increases nodule free oxygen that can directly inhibit Nase gene expression and activity (Becana *et al.*, 2010). Nodule permeability to oxygen through the operation of ODB can also participate in BNF regulation upon changes in the environment (Minchin, 1997).

In nodules of several legumes, many environmental constraints, such as dark stress (Gogorcena *et al.*, 1997), drought (Gogorcena *et al.*, 1995), or excess nitrogen (Escuredo *et al.*, 1996), are known to induce ROS overproduction, causing BNF inhibition. These stresses affect many plant metabolic processes including a decrease of nodule antioxidant capacity as a consequence of oxidative damage that may even lead to cell death. Thus, these stresses provoke a rather generalized harmful effect in the plant which is often associated with a deleterious effect on plant shoot performance. To study oxidative stress-avoiding effects on shoot and oxidative damage, Marino *et al.* (2006) applied a low concentration of paraquat to nodulated pea plants, which provoked an effect on BNF before any other effect on plant performance was evident. The authors suggested that the BNF decline was related to SS down-regulation and to a limitation in carbon supply to bacteroids (Marino *et al.*, 2006).

In this study, we applied a dose of Cd that was low enough to avoid any change in shoot biomass (Table 1) or chlorophyll content (Fig. 2), two of the most commonly accepted markers of Cd toxicity in plant photosynthetic organs (Gallego *et al.*, 2012). The effect of severe Cd treatments on nodule functioning has already been studied in several legumes, for instance in white lupin (Carpena *et al.*, 2003), pigeonpea (Garg and Aggarwal, 2012), and mung bean (Muneer *et al.*, 2012). However, in those studies Cd treatment also affected shoot biomass and/or chlorophyll content. Thus, it is not possible to distinguish whether nodule activity impairment was related to a general plant breakdown or to a direct effect on nodule components. In our study, although approximately 1% of the Cd taken up by ST roots was translocated to both shoots and SC roots, this translocation was at the time of harvest low enough to avoid toxicity in shoots (Table 1, Fig. 2) and to avoid a decline in nitrogen fixation in SC nodules (Fig. 4). Thus, this SRS approach shows that BNF inhibition provoked by Cd is due to a direct effect on nodules rather than a systemic effect through a control from the shoot. Local regulation of nitrogen fixation has already been shown under moderate water stress in pea (Marino *et al.*, 2007) and more recently in *M. truncatula* (Gil-Quintana *et al.*, 2013*a*) and soybean (Gil-Quintana *et al.*, 2013*b*). The present work, with the application of Cd, which is a very mobile element (Lux *et al.*, 2011), confirms the high and local sensitivity of the nitrogen-fixation process to environmental constraints.

To understand the mechanism of this nitrogen-fixation inhibition, we measured lipid oxidation to determine whether Cd was generating oxidative damage in nodules. MDA content was similar in control nodules (C and SC) and in nodules in direct contact with Cd (Fig. 5). Moreover, at the time of harvest root necrosis did not occur (Fig. 1C). Thus, in our conditions, the hypothesis that BNF inhibition was due to a generalized collapse of the nodule can be discarded. Furthermore, total nodule protein content was similar in C, SC, and ST nodules (data not shown). Although several studies have already studied Cd toxicity on *M. truncatula* shoot and root biology (Xu *et al.*, 2010; Aloui *et al.*, 2011), to our knowledge this work represents the first time that the effect of Cd on legume nodule functioning has been reported.

The induction of antioxidant systems appears to be a key player in the avoidance of severe effects of abiotic stress (Apel and Hirt, 2004; Suzuki *et al.*, 2012). In legume nodules of *M. truncatula* plants overexpressing γ-ECS or in nodules of wild-type plants inoculated with a *S. meliloti* strain overexpressing γ-ECS, an increase in nitrogen-fixation capacity was reported (El Msehli *et al.*, 2011). In common bean it seems that higher antioxidant defences would have a protective role against osmotic stress (Sassi *et al.*, 2008). However, an increase in ascorbate content in pea nodules did not have a protective role against drought (Zabalza *et al.*, 2008). In our work, the expression of antioxidant-enzyme-encoding genes was up-regulated (Fig. 6), including a high induction of γ-ECS (Fig. 6A). Nevertheless, antioxidant machinery induction was not sufficient to prevent BNF inhibition, showing the high sensitivity of this process to environmental fluctuations. Most of the determined antioxidants were induced only in ST nodules, but CAT (from plant or bacteria), ICDH, 6-phosphogluconate dehydrogenase, and FeSOD were also induced in SC nodules, suggesting either a systemic control or a very sensitive response to the slight increase in Cd content that was translocated from ST roots. CAT, a direct scavenger of H_2_O_2_, is one of the major antioxidants in plants (Apel and Hirt, 2004). In this work, CAT expression and activity from the plant side (Figs 6C and 7) and *Katb* expression from the bacterial side (Fig. 6D) were induced both in SC and ST nodules. The heterologous overexpression of a CAT gene from *Brassica juncea* in tobacco plants conferred to these plants an enhanced tolerance to Cd (Guan *et al.*, 2009), limiting oxidative damage, as demonstrated by MDA monitoring. In our work the high induction of CAT might be essential to control generalized nodule oxidative damage but not enough to avoid a decline in BNF.

To gain further knowledge in the regulation of BNF in *M. truncatula* nodules under Cd we measured the transcription and translation of three of the most important components of nodule response to abiotic stress: SS, Lb, and NifD, one of the components of Nase enzymatic complex. SS was not affected by the stress; however, both Lb and *NifD* gene expression and protein content decreased. This is consistent with other studies on Cd treatment, in soybean (Balestrasse *et al.*, 2004) or common bean (Loscos *et al.*, 2008), where a decline in nitrogen fixation under Cd has been associated with a decrease in Lb content. However, in those studies Cd stress was very severe and oxidative damage was evident together with a down-regulation of antioxidant machinery. In our study, nodule antioxidant systems were still induced upon Cd exposure and could avoid generalized damage. However, as stated, Lb is a very sensitive protein to redox changes and its content dramatically declined in ST nodules. The function of this globin is to supply oxygen to the bacteroids, to support their energy-yielding respiration, at a tension low enough to avoid disrupting Nase, which is highly sensitive to oxygen. Thus, a Lb decline will provoke an alteration of the nodule microaerophilic environment that in turn will lead to the down-regulation of Nase expression and content (Fig. 8A, B), which ultimately will result in inhibition of nitrogen fixation (Fig. 5). ODB may also play a role in the control of nodule oxygen concentration to regulate BNF (Minchin, 1997). Lb degradation could cause a transient increase in nodule internal oxygen that could provoke ODB closure. Moreover, ROS have been also suggested to regulate ODB, although direct experimental evidence is still lacking (Minchin, 1997; Minchin *et al.*, 2008). Thus, the interaction Lb–ROS–O_2_–ODB may be responsible for BNF regulation.

The heterologous overexpression of a flavodoxin from *Anabaena variabilis* in *S. meliloti* partially prevented Cd toxicity effects on Nase activity in *Medicago sativa* (Shvaleva *et al.*, 2010). Flavodoxins are prokaryotic electron carrier proteins and have been suggested to play a positive role in ROS detoxification (Redondo *et al.*, 2009). This result is in agreement with our data indicating that the effect of Cd on nitrogen-fixation capacity in *M. truncatula* nodules is related to a down-regulation of Nase expression and protein content, rather than SS down-regulation. In *M. sativa* drought-stressed plants, nitrogen fixation was suggested to be related to a direct effect on Nase (Naya *et al.*, 2007). In that work, however, drought treatment had already provoked generalized oxidative damage (Naya *et al.*, 2007).

Taken together, our data reveal that the application of moderate Cd treatment in SRS-grown *M. truncatula* plants provoked mild stress where BNF partial inhibition was controlled at the local level. We suggest that this control is exerted through Lb/oxygen availability that affects Nase function rather than being an effect provoked by a general breakdown of plant cells due to a severe oxidative damage. Interestingly, it seems that in forage legumes, which include *M. sativa* and *M. truncatula* species, mild stress provokes a control of nitrogen fixation related to Lb/oxygen availability rather than having a rapid effect on sucrose synthase, as is known in grain legumes such as pea, bean, or soybean (Arrese-Igor *et al.*, 2012). Thus, it appears that control of nitrogen fixation is not only dependent on the type and severity of the stress but also in the legume species. Future studies using plants with altered ROS-scavenging and/or -producing mechanisms might be useful to advance our understanding of the duality of the signalling/toxic role of ROS in nitrogen-fixation regulation and in plant responses to environmental fluctuations. Besides, it is still controversial whether ROS can travel long distances in the plant, since most of them are highly reactive. Miller *et al.* (2009) proposed ROS as important players in systemic signals propagation in *Arabidopsis*.

## Supplementary material

Supplementary material is available at *JXB* online.

Table S1. List of primers used to quantify gene-expression levels by quantitative PCR.

Supplementary Data

## Abbreviations:

APX: ascorbate peroxidase
BNF: biological nitrogen fixation
C: control
CAT: catalase
γ-ECS: γ-glutamyl-cysteine synthetase
GPX: guaiacol peroxidases
GR: glutathione reductase
ICDH: NADP^+^-dependent isocitrate dehydrogenase
Lb: leghaemoglobin
MDA: malondialdehyde
MR: monodehydroascorbate reductase
Nase: nitrogenase
ODB: oxygen diffusion barrier
ROS: reactive oxygen species
SC: split-control
SOD: superoxide dismutase
SRS: split-root system
SS: sucrose synthase
ST: split-treatment
T: treatment.
