# Supplementary material for: Inhibition of nitrogen fixation in symbiotic Medicago truncatula upon Cd exposure is a local process involving leghaemoglobin
Source: J Exp Bot. 2013 Oct 22;64(18):5651–60. doi: 10.1093/jxb/ert334 (PMC3871818; doi:10.1093/jxb/ert334)
Supplement: Supplementary Data [file supp_64_18_5651__index.html]

Inhibition of nitrogen fixation in symbiotic Medicago truncatula upon Cd exposure is a local process involving leghemoglobin — Inhibition of nitrogen fixation in symbiotic Medicago truncatula upon Cd exposure is a local process involving leghaemoglobin — Supplementary Data 

# Inhibition of nitrogen fixation in symbiotic *Medicago truncatula* upon Cd exposure is a local process involving leghaemoglobin

## Supplementary Data

Data files

**Files in this Data Supplement:**

- Supplementary Data - Supplementary Data
