# Supplementary material for: Inhibition of nitrogen fixation in symbiotic Medicago truncatula upon Cd exposure is a local process involving leghaemoglobin
Source: J Exp Bot. 2013 Oct 22;64(18):5651–60. doi: 10.1093/jxb/ert334 (PMC3871818; doi:10.1093/jxb/ert334)
Supplement: Supplementary Data [file supp_ert334_jexbot107227_file001.pdf]

Table S1. List of primers used to quantify gene expression levels by qPCR.

Gene	Forward primer	Reverse primer
Medicago truncatula		
MtγECS	CTAGAGCTGGCAAAGGATGG	CTCTCAGCTGGAGTGACACCT
GSHS	CAGTGCGAGACACCCTCATA	GCAACCACCATTACGCATC
hGSHS	CCTCACATGATGAAACCAACTC	CAAGCAGGTCGCTGTCTCTT
GalLDH	ATTGGGCTTTGATTGTGGAG	AATTGGAGCAGGAGCAGGTA
ICDH	TTTCAGGGTTCACCAAAAGG	CCAAGAGCGTAGCATTGTCA
G6PDH	GCAACCTGGACTGGAAATGT	GCTCGTAAGCCTCTGGAATG
6PGDH	TTGCTGAGGCTTACGATGTG	TTCTCCCTTGTTCCATTCGG
FeSOD	CGGAGAGGGAGAAAGAGGAG	TTCTAGTCGGTATCACTATCTGC
MnSOD	GCTCTTCAAGGGTCTGGATG	CCAAGCAATGGAACCAAACT
CuZnSODp	ATGGTTTTCACCTCCACGAG	CGTTGGCATCAGCAATTATG
CuZnSODc	TGGGAAAGGTGGTCATGAGC	ACAAGCTACTCTGCCACCAGC
CAT	GAGCAAGTGCAAAGGGTTTC	ATGACGGGTGTCTGAACTCC
MR	CTGTTGCTACTGGATTCACCTC	CTCCCAACACCAACAACAAC
GR	GAAGCCAGATTATAGGGATATTC	ATAAATCGCCGTTTGTTTGC
Lb	ATAGCTCATATGAGGCATTCAAA	GAGTTGAGGACTATCTTGTACT
SS	ATTCAACTTGTAGGTCTCGAT	GTTGAGAATGAAGAGCACATA
Mtc27	TTACTCGAGAGTTGAGACTATG	TCAAACCTGACAACTGACATTC
40S ribosomal protein	TTGTCCAAGTTTGATGCTGCAC	TCCTACCAACTTCAAAACACCG
Sinorhizobium meliloti		
SmKatA	GGGACTACTGGTCCCTGTCG	TCAGGATCGTCACCTGATGC
SmKatB	AGCCCTATCCACCAGAGCCT	ACCACCCGTCAGCAATTCAT
SmKatC	GACCTCGTCGGGAACAACAT	CTTGATTGCGTCCTGGATGA
SmSodA	GTGCCTATGTTCCGCTGACA	TCCTCTTGGCATGGATTTCG
SmSodC	CGTGCTGATCGAGATGGAGA	GCCACCCAGGTATCCTTCG
SmNifD	GCAAAGCTAAATCTTATCCAC	AGGTCCTCATAGGCAGTAATA
Smc00324	GGACGGCTACAAGAACGACAC	TCATGCTGCATGACCGTGAT
rRNA 16S	GATAAGCCGAGAGGAAGGTG	GTGTAGCCCAGCCCGTAAG
